# The Impact of Hippocampal Sex Hormones Receptors in Modulation of Depressive-Like Behavior Following Chronic Anabolic Androgenic Steroids and Exercise Protocols in Rats

**DOI:** 10.3389/fnbeh.2019.00019

**Published:** 2019-02-07

**Authors:** Dragica Selakovic, Jovana Joksimovic, Nemanja Jovicic, Slobodanka Mitrovic, Vladimir Mihailovic, Jelena Katanic, Dragan Milovanovic, Suzana Pantovic, Natasa Mijailovic, Gvozden Rosic

**Affiliations:** ^1^Department of Physiology, Faculty of Medical Sciences, University of Kragujevac, Kragujevac, Serbia; ^2^Department of Histology and Embryology, Faculty of Medical Sciences, University of Kragujevac, Kragujevac, Serbia; ^3^Department of Pathology, Faculty of Medical Sciences, University of Kragujevac, Kragujevac, Serbia; ^4^Department of Chemistry, Faculty of Science, University of Kragujevac, Kragujevac, Serbia; ^5^Department of Pharmacology and Toxicology, Faculty of Medical Sciences, University of Kragujevac, Kragujevac, Serbia

**Keywords:** anabolic androgenic steroids, exercise, depression, androgen receptors, estrogen α receptors, hippocampus, rats

## Abstract

The aim of this study was to evaluate alterations in depressive-like behaviors in rats following chronic administration of a supraphysiological dose of anabolic androgenic steroids (AASs) as well as exposure to a prolonged exercise protocol. The role of hippocampal sex hormones receptors in the modulation of depressive-like behavior was also assessed. A total of 48 male Wistar albino rats were divided into six groups: control, exercise (1 h/day, five consecutive days), nandrolone-decanoate (ND, 20 mg/kg/week, in a single dose), exercise plus ND, testosterone-enanthate (TE, 20 mg/kg/week, in a single dose), and exercise plus TE. After the 6-week protocols were complete, the rats underwent behavioral testing in the tail suspension test (TST). Rats were sacrificed for the collection of blood samples, to determine sex hormones levels, and isolation of the hippocampus, to determine [androgen receptors (AR) and estrogen receptors α (ERα)] expression. ND and TE treatment induced significant depressive-like behavior, opposing the antidepressant effect of exercise. Chronic TE administration elevated testosterone (T) and dihydrotestosterone (DHT) serum levels, and this was augmented by exercise. In contrast, ND and exercise alone did not alter T or DHT levels. There were no changes in serum estradiol levels in any of the groups. Immunohistochemical analysis showed that exercise reduced AR immunoreactivity in all hippocampal regions and increased the ERα expression in the CA1, dentate gyrus (DG), and total hippocampal sections, but not in the CA2/3 region. AASs administration increased AR expression in all hippocampal regions, although not the total hippocampal section in the TE group and did not significantly decrease ERα. The hippocampal AR/ERα expression index was lowered while parvalbumin (PV)-immunoreactivity was enhanced by exercise. AASs administration increased the AR/ERα index and reduced PV-immunoreactivity in the hippocampus. The number of PV-immunoreactive neurons negatively correlated with the antidepressant effects and the AR/ERα ratio. Our results suggest a potential role of the numerical relationship between two sex hormones receptors (stronger correlation than for each individual receptor) in the regulation of depressive-like behavior *via* the hippocampal GABAergic system in rats, which allow better understanding of the hippocampal sex hormones receptors role in modulation of depressive-like behavior.

## Introduction

Anabolic androgenic steroids (AASs) represent a large group of synthetic derivatives of testosterone. Although there are several clearly defined clinical indications for the therapeutic use of AASs, misuse and even abuse of these compounds has existed for decades. The abuse of AASs involves the long-term use of different types of synthetic steroids at supraphysiological doses, which results in a wide range of side effects, including various psychiatric manifestations. The appearance and frequency of adverse effects depend on the doses used, the duration of use, as well as the individual characteristics of users and environmental factors. Behavioral manifestations induced by AASs abuse include the occurrence of frequent mood disorders, aggression, manic symptoms, and paranoid jealousy (Pope and Katz, 1988; van Amsterdam et al., 2010). The most common adverse reactions to AASs abuse are depression and suicide (Thiblin et al., 1999). Furthermore, it has been found that depression may even appear as a consequence of withdrawal (Gruber and Pope, 2000). In addition, numerous reports have confirmed AASs-induced depressive-like behavior in experimental animal models (Matrisciano et al., 2010; Tucci et al., 2012).

AASs abuse, among adolescents in particular, is commonly accompanied by significant lifestyle alterations, such as various training programs, as well as strict dietary regimes. Various physical activity protocols have significant beneficial effects on the brain by means of improving mental health and represent a preventive method for many psychotic disorders. Previous articles have also shown that exercise induces an increase in cerebral blood flow, brain volume, and improves cognitive abilities in humans (Hopkins et al., 2012; Coelho et al., 2013) and rodents (van Praag et al., 1999). One of the most robust and sustained mechanisms involved in the exercise-induced antidepressant effect is the elevation of hippocampal brain-derived neurotrophic factor (BDNF) mRNA levels during voluntary exercise in normal rats (Oliff et al., 1998; Molteni et al., 2000) and also in an animal model of depression (Zheng et al., 2006). However, the antidepressant effect of exercise is, in general, still speculative. Although it was observed that acute exercise of any intensity significantly improved depressive symptoms in women (Meyer et al., 2016), other sources have shown that correlations between physical activity and depression depend on the intensity of the exercise (Noh et al., 2015). After all, the results of study showing the most beneficial behavioral effects of exercise, including antidepressant effects, were achieved by using prolonged exercise protocols with mild (erobic) intensity (Joksimović et al., 2017b).

The evaluation of behavioral alterations, following the protocols mentioned above, usually includes the analysis of events that require the activity of specific brain regions involved in mood regulation, such as the hippocampal region. The hippocampus is a brain region that plays a key role in cognitive and emotional processing. It contains two basic groups of neurons, i.e., principal neurons, responsible for connection to other brain structures, and interneurons, which are predominantly GABAergic, that form the hippocampal neuronal network (Campbell and Macqueen, 2004). GABAergic interneurons are widespread throughout various brain regions and play an important role in the modulation of local noradrenergic, dopaminergic, serotonergic, and glutamatergic neuronal circuits. GABAergic system dysfunction may be responsible for the appearance of depressive symptoms (Holm et al., 2011), in various mood disorders (Brambilla et al., 2003) and bipolar disorder (Heckers et al., 2002), as well as for the pathogenesis of post-traumatic stress syndrome (Liu et al., 2016). The GABAergic interneurons in the hippocampus can be divided, according to specific immunoreactivity, into the following subpopulations: neuropeptide Y-, somatostatin-, dynorphin-, and parvalbumin (PV)-positive interneurons. PV is classified as a calcium-binding protein that is specifically found in vertebrates (Schwaller et al., 2002), and it is primarily found in the hippocampus (Zaletel et al., 2016). Since the activity of hippocampal GABA_A_ receptors is considered to be a crucial factor in mood regulation, including anxiety and depression, it appears that the AASs-induced allosteric modification of these receptors may be an important mechanism in behavioral control (Möhler, 2012).

The evaluation of sex hormones-induced behavioral alterations must involve the analysis of the interconnection between the elements of this system (receptors, metabolism, etc.) and the systems that are directly employed in mood regulation. Sex hormones receptors, namely the androgen receptors (ARs), and estrogen receptors α and β (ERα, β), are present in almost all brain regions, including those involved in behavioral control, such as the hippocampus (Menard and Harlan, 1993). Within the hippocampus, ERα has been localized to pyramidal neurons (Milner et al., 2001) and interneurons, both inhibitory (GABAergic; Rai and Jeswar, 2012) and excitatory (cholinergic; Towart et al., 2003). The localization of sex hormones receptors in hippocampal neurons is quite heterogeneous, i.e., nuclear and extranuclear (including the cell membrane, mitochondria, and synaptic vesicles; Tabori et al., 2005). Beside neurons, sex hormones receptors are also found in oligodendrocytes and astroglia, marking those cells as target sites for the action of steroids as well (Jung-Testas and Baulieu, 1998; Finley and Kritzer, 1999). Interestingly, the highest level of AR and ER co-localization in rodents was observed in the amygdalohippocampal area (Wood and Newman, 1995). Brain regions involved in behavioral regulation not only contain sex hormones receptors, but also show the largest content of enzymes essential for steroids synthesis and biotransformation (Roselli et al., 2009). The significant role of sex hormones receptors in the control of aggressive behavior was previously evaluated in mice with a genetic modification of AR (Robinson et al., 2012). However, similar behavioral manifestations were observed through interventions on ERα (Scordalakes and Rissman, 2003) due to the signaling shift of AASs that appears as a consequence of the AASs-induced modification of aromatase activity in the brain (Roselli et al., 2009). In addition, the impact of AASs son the control of anxiety and aggressive behavior may be achieved in a way that does not involve sex hormones receptors, but instead through alterations in the biosynthesis of endogenous neurosteroids (Pinna et al., 2005, 2008). However, the impact of sex hormones on their hippocampal receptors may not be evaluated without assuming that local synthesis in the hippocampus results in the significant differences in hippocampal-synthesized and circulation-derived sex steroids (Hojo et al., 2009) that can be ascribed to the different activities of sex steroidogenesis-related enzymes (P450 aromatase and 5α reductase) in the brain (Hojo et al., 2004).

The aim of this study was to evaluate the alterations in depressive-like behavior following chronic administration of AASs at a supraphysiological dose, as well as following a prolonged exercise protocol. The expression of sex hormones receptors in rat hippocampus was also assessed. In addition, we analyzed the numerical relationship between hippocampal AR and ERα in a bid to ascertain the histological algorithm that may underlie the behavioral alterations.

## Materials and Methods

All research procedures were carried out in accordance with European Directive for welfare of laboratory animals No 86/609/EEC and principles of Good Laboratory Practice (GLP), and according to ARRIVE guidelines. All experiments were approved by Ethical Committee of the Faculty of Medical Sciences, University of Kragujevac, Serbia.

A total of 48 male Wistar albino rats (3 months old, 350–400 g), housed in groups of four per cage, under controlled environmental conditions (temperature — 23 ± 1°C and light — 12/12 h light/dark cycle), were used in the study. The rats had free access to food and water. Animals were divided into six groups (eight rats per group), as follows: control (C), exercise (E), nandrolone-decanoate (ND), exercise plus ND (E+ND), testosterone-enanthate (TE), exercise plus TE groups (E+TE). TE and ND were chosen as two of the most commonly abused AASs that belong to different AASs classes (testosterone esters and derivatives of 19-nortestosterone, respectively). All animals were continuously monitored by a veterinarian on a daily basis for general health (with no visible signs of morbidity), and food and water intake, while the body weight was recorded weekly, with no significant difference noted between the groups throughout the trial.

The ND groups (ND and E+ND) and TE groups (TE and E+TE) received 20 mg/kg subcutaneous injections of AASs (DEKA 300, SteroxLab, EU and Testosteron depo, Galenika a.d., Serbia, respectively) weekly, in a single dose, for 6 weeks. The final doses of ND and TE were chosen to mimic the doses for heavy human AASs abusers (Kanayama et al., 2008), since these were confirmed to be sufficient to induce behavioral alterations in our previous studies (Selakovic et al., 2016, 2017; Joksimović et al., 2017a,b). The exercise protocols also lasted for 6 weeks (in groups E, E+ND, and E+TE). For this protocol, rats swam for 1 h per day, for five consecutive days with 2 days break in a glass tank (60 × 75 × 100 cm, 60 cm water depth, heated at 32 ± 1°C), in groups of four. To reduce water-induced stress, without promoting physiological alterations related to physical training, the familiarization of animals with water was achieved by immersion (15 min daily for 1 week) in a tank with a water depth of 7 cm before the initiation of the exercise protocol (Contarteze et al., 2008). Swimming sessions were chosen as a suitable exercise protocol since swimming is considered a natural (inherited) behavioral pattern among rodents (Sugizaki et al., 2006). The exercise protocol properties (the duration of the single trial and complete protocol, as well as recovery periods) were selected according to the reported results for the effects of swimming protocols on behavioral alterations accompanied by morphological changes in rat hippocampus (Joksimović et al., 2017a; Selakovic et al., 2017). Both combined groups (E+ND and E+TE) simultaneously underwent AASs administration and exercise protocols for 6 weeks. Rats from the sedentary groups (C, ND, and TE) were placed in the same water tank for 2 min each day of the training protocol in order to minimize the difference between the exercise and non-exercise groups caused by a reaction to water immersion. With the aim of maintaining the same social context of the exercise protocol, sedentary rats were also put in water in groups of 4 animals. Swimming sessions were continuously monitored by an experimenter during the entire duration of the swimming task. In order to avoid floating, which that could seriously alter the level of exercise, as soon as it was observed, the experimenter immediately pulled the floating down animal by 5–10 cm in the water by the tail when this occurred. After completing each swimming session, rats were towel dried and placed in a clean cage. In order to eliminate differences between the groups during the pre-treatment, the animals that did not receive AASs (C and E groups) underwent parenteral treatment with sterilized olive oil, using the same volume and route of administration.

In order to retain algorithms established during pre-treatment, 2 days after completing the described protocols, the rats were allowed to acclimate in a testing room (approximately at 8 am) for at least 1 h before the start of the behavioral testing with the tail suspension test (TST), following the rule that the same-housed animals had to be tested on the same day (starting at approximately 9 am).

### Tail Suspension Test (TST)

The estimation of depressive-like behavior was performed using the TST for 6 min. This test is based on the assumption that an animal will actively try to escape an aversive (stressful) stimulus (Chermat et al., 1986). The test was performed according to a previously described procedure (Joksimović et al., 2017b). The whole test trial was recorded using a video camera, and recordings were analyzed in order to determine the following parameters (data presented in Supplementary Table S1): the latency to the first immobility, the number of immobility episodes, the total duration of immobility (TDI) and the average duration of an immobility episode (the ratio of the TDI to the number of immobility episodes). Testing was performed under proper conditions of silence and illumination for this type of behavioral testing (the room was illuminated with controlled light, ~100 lx) as previously described (Selakovic et al., 2017).

Immediately following behavioral testing, the animals were anesthetized by short-term narcosis, induced by intraperitoneal application of ketamine (10 mg/kg) and xylazine (5 mg/kg), and then sacrificed by decapitation. Trunk blood samples were collected for determination of serum sex hormones levels, while (simultaneously) brains were rapidly removed from the skull for histological analysis.

### Serum Hormone Assays

Trunk blood was collected and allowed to clot at room temperature for 2 h in anticoagulant-free tubes, and then centrifuged at 3,500 *g* for15 min at 4°C. The clear supernatant was kept at −80°C until analysis. Serum samples were assessed for the determination of sex hormones levels (data presented in Supplementary Table S1): total testosterone (T), dihydrotestosterone (DHT), and estradiol (E2) levels. T and E2 levels were determined by Elecsys 2010 analyzer using the method of the electrochemiluminescence immunoassay (ECLIA). Standard commercial kits (Elecsys Testosterone II and Estradiol III, Roche Diagnostics, Mannheim, Germany) were used and the T and E2 levels were expressed in ng/ml and pg/ml, respectively. The sensitivities of the assays for T and E2 were 0.025 ng/ml and 5 pg/ml, respectively. Inter- and intra-assay coefficients of variance for T and E2 were 3.8% and 2.2%, and 5% and 3.9%, respectively. Serum DHT levels were measured using a sensitive kit (ALPCO Diagnostics, Salem, NH, USA) using the ELISA method and the values were expressed in pg/ml. The sensitivity of the assay for DHT was 6.0 pg/ml. Inter- and intra-assay coefficients of variance for DHT were 5.9% and 3.9%, respectively.

### Immunohistochemistry

Immediately after decapitation, rat brains were carefully and gently removed and then a previously described procedure (Joksimović et al., 2017b) was followed:-fixation in 4% neutral buffered formaldehyde, dehydration, and embedding. Coronal brain sections, 5 μm thick were dewaxed, rehydrated and treated with citrate buffer (pH 6.0) in the microwave for antigen retrieval. Endogenous peroxidase activity was blocked with 3% H_2_O_2_, and non-specific labeling was blocked using a commercial protein block (Novocastra, UK). Slices were incubated with the primary antibody mouse monoclonal anti-PV (1:1,000, Sigma-Aldrich) overnight at room temperature. Labeling was performed using a biotin-conjugated secondary antibody, followed by streptavidin-HRP, and visualization was performed with 3,3′-diaminobenzidine (DAB) chromogen (Peroxidase Detection System RE 7120-K, Novocastra, UK). After that, the sections were counterstained with Mayer’s hematoxylin and covered. For AR staining, formalin-fixed paraffin-embedded (FFPE) sections were incubated with AR Monoclonal Antibody (4 μg/ml, AN1–15, MA1–150, Thermo Fisher scientific, Carlsbad, CA, USA), overnight at room temperature. The slides were then incubated with secondary Rabbit Anti-Rat IgG H&L (1:1,000, ab6703, Abcam, UK) for 30 min. An EXPOSE Rabbit specific HRP/DAB detection IHC Kit (ab80437, Abcam, UK) was used according to the manufacturer’s protocol. For ERα staining, the slides were incubated with Anti-ER alpha antibody (E115; 1:200, ab32063, Abcam, UK) overnight at room temperature. An EXPOSE Rabbit specific HRP/DAB detection IHC Kit (ab80437, Abcam, UK) was used according to the manufacturer’s protocol. All primary antibodies were diluted in Antibody Diluent (003118, Thermo Fisher scientific, Carlsbad, CA, USA).

**Figure 1:**
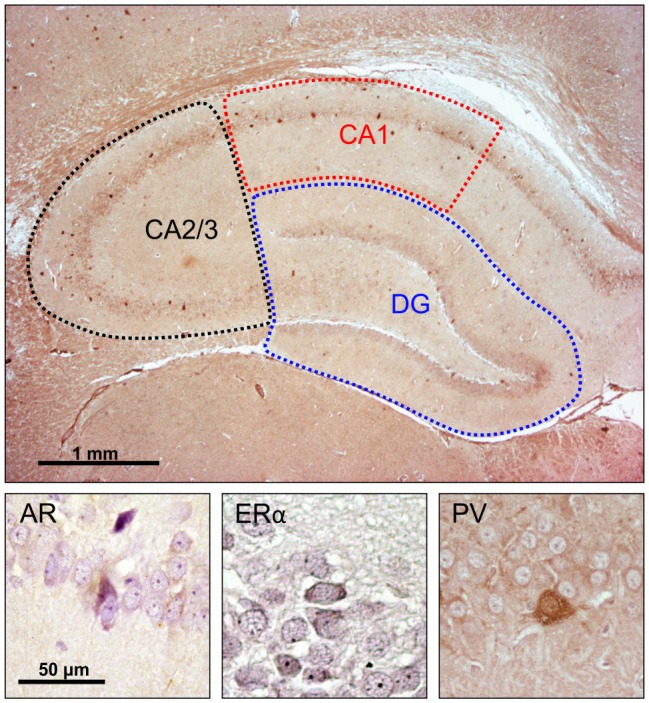
Immunohistochemical analysis of the rat hippocampus. Upper panel: investigated hippocampal regions (microphotography obtained with specific parvalbumin (PV) immunostaining). Lower panel, immunoreactivity for: androgen receptors (ARs; left), estrogen receptors α (EAα; center), PV neurons (right).

Microscopy was performed with a light microscope (Carl Zeiss, Axioscop 40), and images for representative fields were captured using the camera (Canon PC 1089) and AxioVision 4.7 software system. The assessment of hippocampal immunoreactive cells was performed unilaterally (alternately in the left or right hemisphere) for all animals. The number of immunoreactive cells was always obtained on the dorsal hippocampus (the level of the section was 3.8 mm caudal to Bregma, according to the Paxinos and Watson, 1998 stereotaxic atlas) on one series (5–7) of hippocampal sections per animal, and expressed as an average per 1 mm^2^ of the investigated hippocampal region [CA1, CA2/3 and dentate gyrus (DG)], as previously described (Selakovic et al., 2017). Also, the total number of immunoreactive cells in the whole hippocampal sections was calculated as the sum of immunoreactivity obtained in the total surface of all three investigated hippocampal regions (Figure 1). Two independent experimenters who performed the counts (presented in Supplementary Table S1) were blind to the sample classification and showed high inter-rater reliability for AR, ERα and PV counts (Pearson’s *r* = 0.924, 0971, and 0.975, respectively).

The quantitative relationship between AR and ERα expression in hippocampal sections was calculated as the quotient of the numbers of immunoreactive cells for those two receptors (multiplied by 100) and expressed as the AR/ERα index.

### Statistical Analysis

The data presented herein are expressed as the means ± standard error of the mean (SEM). The data were initially analyzed using Levene’s test for homogeneity of variance and the Shapiro-Wilk test of normality. Comparisons between groups were performed using a one-way analysis of variance (ANOVA), followed by Bonferroni *post hoc* analysis. To analyze the independent and joint effects of two different independent variables (exercise and AASs) on investigated outcomes in the groups with significant differences, we performed a two-way ANOVA (data presented in Tables A–C, in Supplementary Table S2). Pearson’s coefficient of correlation was used to analyze the relationships between parameters, and simple linear regression analyses were performed. Differences with *p* < 0.05 were considered to be statistically significant. Statistical analysis was performed with SPSS version 20.0 statistical package (IBM SPSS Statistics 20).

## Results

### Opposing Effects of AASs and Exercise on Depressive-Like Behavior

**Figure 2:**
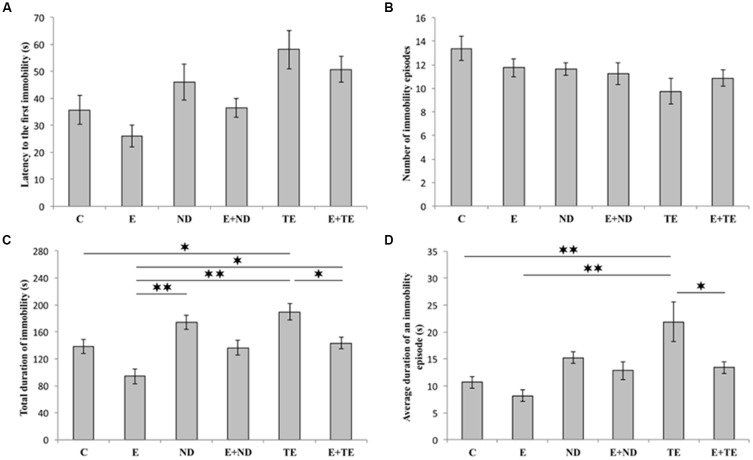
Parameters obtained in the tail suspension test (TST): **(A)** The latency to the first immobility, **(B)** the number of immobility episodes, **(C)** the total duration of immobility (TDI), **(D)** the average duration of an immobility episode. C, control; E, exercise; ND, nandrolone-decanoate; E+ND, exercise plus ND; TE, testosterone-enanthate; E+TE, exercise plus TE group [Mean ± standard error of the mean (SEM), *n* = 8 per group, *denotes a significant difference *p* < 0.05, **denotes a significant difference *p* < 0.01].

As shown in Figure 2, the applied protocols resulted in significant alterations in depressive-like behavior as indicated by the parameters obtained in TST. However, latency to the first immobility (Figure 2A) and the number of immobility episodes (Figure 2B) did not significantly change following the described chronic protocols (*F* = 2.877 and 1.927, respectively; *df* = 5). In contrast, a prolonged exercise protocol, as well as administration of AASs, induced opposite effects on the TDI (*F* = 10.103). The depressive-like effect of chronic administration of AASs was manifested by increased TDI (*p* < 0.05, for TE). Conversely, physical activity resulted in a significant antidepressant effect compared with the administration of both AASs (*p* < 0.01), although with no significant decrease in TDI compared to control group (Figure 2C). The antidepressant effect of exercise was additionally confirmed by decreased TDI in the combined groups compared with the sedentary AASs groups (*p* < 0.05, for TE). The strong depressive-like effect of chronic TE administration (*F* = 6.154) was also demonstrated by alterations in the average duration of an immobility episode (Figure 2D). The level of this indicator of increased depressive-like behavior was significantly higher in the sedentary TE group than in both control and exercise groups (*p* < 0.01), as well as the combined TE group (*p* < 0.05). Further statistical analysis (Table A in Supplementary Table S2) also showed that both exercise and AASs administration protocols significantly (*p* < 0.01) affected TDI, with almost equal effect sizes (η^2^ = 0.374 and 0.375, respectively), as well as the average duration of an immobility episode (*p* < 0.01), with a stronger effect of AASs administration (η^2^ = 0.165 and 0.312, respectively).

### Increase in the Serum Levels of Sex Hormones Following Chronic Protocols

**Figure 3:**
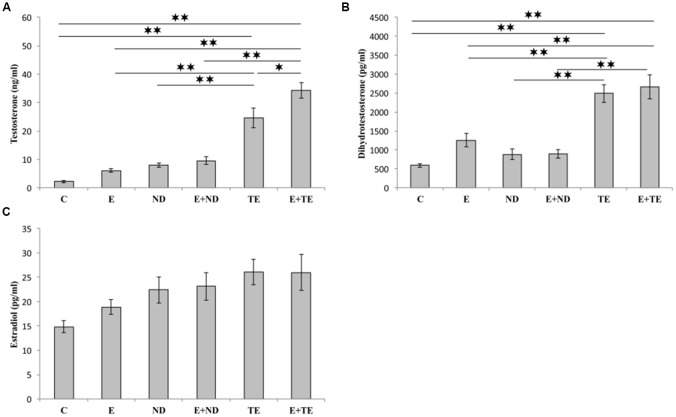
Sex hormones [testosterone **(A)**, dihydrotestosterone (DHT, **B**), and estradiol **(C)**] serum levels. C, control; E, exercise; ND, nandrolone-decanoate; E+ND, exercise plus ND; TE, testosterone-enanthate; E+TE, exercise plus TE group. (Mean ± SEM, *n* = 8 per group, *denotes a significant difference *p* < 0.05, **denotes a significant difference *p* < 0.01).

The applied chronic protocols induced significant alterations in the serum T (*F* = 42.110, *df* = 5) and DHT (*F* = 21.429) levels (Figure 3), with no change in the E2 levels (*F* = 2.844). Treatment with the supraphysiological dose of TE resulted in a significant increase in serum T levels compared with those of the control and exercise groups (*p* < 0.01). The TE-induced rise in serum T levels was observed even compared to ND treated rats (*p* < 0.01). Although the exercise protocol, alone and in combination with ND, failed to induce significant augmentation compared with the control and ND sedentary groups, the exercise performed simultaneously with TE administration resulted in an additional increase in serum T levels compared with those of sedentary TE group (*p* < 0.05).

Similar alterations were observed in the serum DHT levels following chronic protocols of AASs administration and exercise, confirming the dominant effect of TE. The only difference from the serum T levels was noted in the lack of significant additive effects of TE and exercise. The analysis of the effects of the two independent factors (exercise and AASs) on sex hormones levels (Table B in Supplementary Table S2) revealed that both protocols significantly altered (*p* < 0.01) the T levels with a notably stronger effect of AASs (η^2^ = 0.198 and 0.823, respectively), but the serum DHT levels were significantly affected only by chronic administration of AASs (*p* < 0.01, η^2^ = 0.706).

### Opposing Effects of AASs and Exercise on the Expression of Sex Hormones Receptors in the Hippocampus

**Figure 4:**
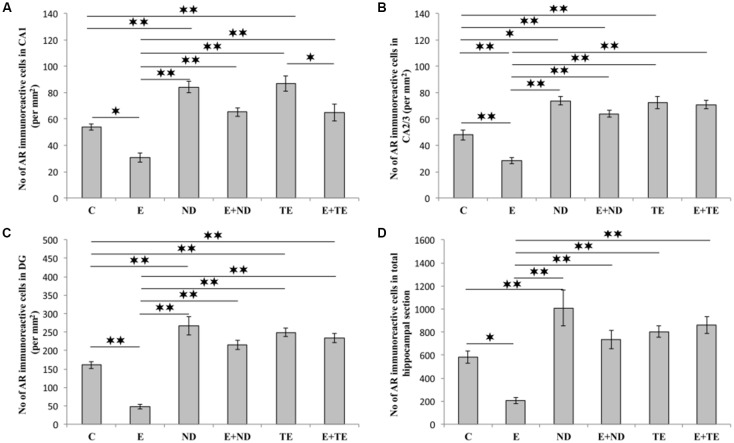
Immunohistochemical expression of ARs immunoreactive cells in rat hippocampal regions: **(A)** CA1, **(B)** CA2/3, **(C)** dentate gyrus (DG), **(D)** total estimated hippocampal section. C, control; E, exercise; ND, nandrolone-decanoate; E+ND, exercise plus ND; TE, testosterone-enanthate; E+TE, exercise plus TE group. (Mean ± SEM, *n* = 8 per group, *denotes a significant difference *p* < 0.05, **denotes a significant difference *p* < 0.01).

All applied protocols resulted in significant alterations in the number of AR-immunoreactive cells in the hippocampus (Figure 4). The opposite effects of exercise and AASs administration protocols were obvious in all the investigated hippocampal regions: the CA1 (*F* = 21.418, *df* = 5), CA2/3 (*F* = 27.980), and DG (*F* = 33.497), as well as in total hippocampal sections (*F* = 13.866). Chronic treatment of sedentary animals with supraphysiological doses of ND and TE induced a significant increase in the number of AR-immunoreactive cells in all the individual regions (*p* < 0.01) and also in the total hippocampal sections (*p* < 0.01 for ND) compared with the control group. Prolonged physical activity significantly decreased AR-immunoreactivity in the investigated hippocampal regions (*p* < 0.05 in the CA1, and *p* < 0.01 in the CA2/3 and DG), as well as in the total hippocampal section (*p* < 0.05), compared with the control and sedentary AASs groups (*p* < 0.01). Further, the exercise-induced decrease in AR-immunoreactivity was confirmed by the lower number of AR-immunoreactive cells in the CA1 of the TE combined group than that of the sedentary TE group (*p* < 0.05). Two-way ANOVA (Table C in Supplementary Table S2) showed that both exercise and AASs administration protocols significantly (*p* < 0.01) affected the number of AR-immunoreactive cells in all the investigated hippocampal regions, as well as on the total hippocampal sections; however, the effect size of the chronic AASs administration protocol was evidently stronger in all the investigated regions (η^2^ values ranging from 0.563 for total section to 0.753 for the DG) than the effects of prolonged swimming exercise (η^2^ values ranging from 0.203 for the total sections to 0.449 in the CA1).

**Figure 5:**
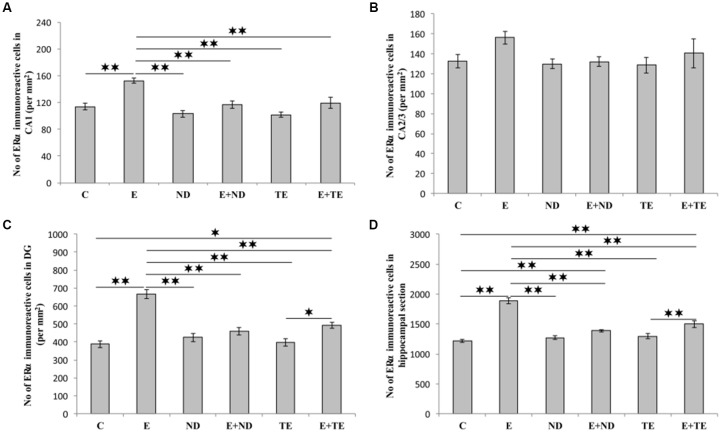
Immunohistochemical expression of estrogen receptors α (ERα) immunoreactive cells in rat hippocampal regions: **(A)** CA1, **(B)** CA2/3, **(C)** DG, **(D)** total estimated hippocampal section. C—control, E—exercise, ND—nandrolone-decanoate, E+ND—exercise plus ND, TE—testosterone-enanthate, E+TE—exercise plus TE group. (Mean ± SEM, *n* = 8 per group, *denotes a significant difference *p* < 0.05, **denotes a significant difference *p* < 0.01).

As shown in Figure 5, the chronic protocols applied in this study also resulted in alterations in the number of ERα immunoreactive cells in the hippocampus, except in the CA2/3 region (Figure 4B), where the immunoreactivity remained the same as that observed in the control group (*F* = 1.569, *df* = 5). While the administration of both AASs in the sedentary groups had no effect on ERα-immunoreactivity compared with the control group, the prolonged exercise protocol resulted in a significant increase in ERα-immunoreactivity in the CA1 (*F* = 10.757), DG (*F* = 25.456), and total hippocampal section (*F* = 50.891). The exercise-induced reduction of ERα-immunoreactivity was also significant compared with the AASs groups (*p* < 0.01). However, prolonged physical activity was sufficient to increase the number of ERα-immunoreactive cells only in the DG (*p* < 0.05) and whole hippocampal section (*p* < 0.01) in the combined TE group compared with the sedentary TE group. Further analysis of hippocampal ERα-immunoreactivity (Table C in Supplementary Table S2) revealed that both types of applied protocols (exercise and AASs administration) significantly (*p* < 0.01) altered the number of ERα immunoreactive cells in the CA1 and DG regions, as well as on the total hippocampal sections. Unlike AR immunoreactivity, the effect size of the exercise protocol on hippocampal ERα-immunoreactivity was stronger (η^2^ values ranging from 0.379 in the CA1 to 0.784 in the total hippocampal section) than that for AASs administration protocols, with η^2^ values ranging from 0.343 in the DG to 0.457 in the total hippocampal sections).

**Figure 6:**
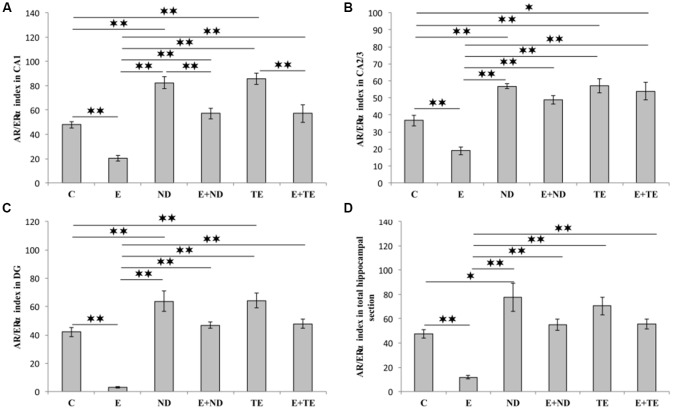
AR/ERα index calculated in rat hippocampal regions: **(A)** CA1, **(B)** CA2/3, **(C)** DG, **(D)** total estimated hippocampal section. C, control; E, exercise; ND, nandrolone-decanoate; E+ND, exercise plus ND; TE, testosterone-enanthate; E+TE, exercise plus TE group. (Mean ± SEM, *n* = 8 per group, *p* < 0.05, ** denotes a significant difference *p* < 0.01).

The AR/ERα index, the parameter introduced in order to allow the estimation of the quantitative (numerical) relationship between AR and ERα in hippocampal sections, augmented the evaluation sensitivity for the analysis of alterations in sex hormones receptors in the rat hippocampus (Figure 6). The AR/ERα index showed that the chronic administration of supraphysiological doses of ND and TE resulted in a significant enhancement (*p* < 0.01) in the ratio of AR to ERα in the CA1 (*F* = 26.992, *df* = 5), CA2/3 (*F* = 20.505), and DG (*F* = 29.068), while this ratio was altered in the total hippocampal section only in ND group (*F* = 13.411, *p* < 0.05). In contrast, the prolonged exercise protocol lowered the AR/ERα index values in all regions, as well as in the total hippocampal section, compared to the control group (*p* < 0.01). The chronic swimming protocol also reduced the AR/ERα index in all regions and the total hippocampal surface compared to the AASs groups (*p* < 0.01). Furthermore, in the CA1, the prolonged exercise protocol resulted in a significant decrease in the AR/ERα index in both combined compared to the sedentary AASs groups (*p* < 0.01). Two-way ANOVA (Table C in Supplementary Table S2) was performed to analyze the effects of the two protocols (exercise and AASs administration) on the numerical relationship between sex hormones receptors (AR-ERα index) expression in the hippocampus. Both factors significantly (*p* < 0.01) altered the AR-ERα index in all investigated hippocampal regions, as well as on the total hippocampal surface. Evidently, the effect size of the chronic AASs protocols was greater in all investigated regions (η^2^ values ranging from 0.499 for the total surface to 0.670 for the DG) compared to the effects of 6 weeks of swimming (η^2^ values ranging from 0.232 for the CA2/3 to 0.552 for the CA1).

**Figure 7:**
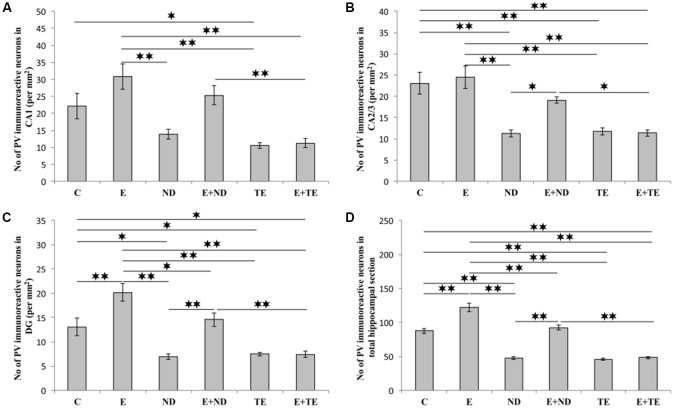
Immunohistochemical expression of PV positive interneurons in rat hippocampal regions: **(A)** CA1, **(B)** CA2/3, **(C)** DG, **(D)** total estimated hippocampal section. C, control; E, exercise; ND, nandrolone-decanoate; E+ND, exercise plus ND; TE, testosterone-enanthate; E+TE, exercise plus TE group. (Mean ± SEM, *n* = 8 per group, *denotes a significant difference *p* < 0.05, **denotes a significant difference *p* < 0.01).

The protocols performed in this study induced significant alterations in the number of hippocampal PV-positive interneurons (Figure 7). Chronic AASs administration reduced hippocampal PV expression in the CA2/3 (*F* = 13.862, *df* = 5), DG (*F* = 17.920), and the total hippocampal section (*F* = 74.746, *p* < 0.01) when compared to the control. In the CA1 region (Figure 7A), only TE application resulted in a significant decrease in PV immunoreactivity (*F* = 10.324, *p* < 0.05). The opposite effect of prolonged exercise manifested as an increased number of PV-positive interneurons when compared to the control, and this effect was observed in the DG and the total hippocampal section (*p* < 0.01), with no significant increase in the CA1 or CA2/3 regions. Interestingly, the exercise-induced augmentation of PV immunoreactivity was also observed between the sedentary and the combined groups (in the DG and the total section of the hippocampus), but only in the ND treated groups. The increased number of PV interneurons in the combined ND group was also significant compared to the combined TE group (*p* < 0.01, except in the CA2/3 where *p* < 0.05). Analyzing the effects of the exercise and AASs protocols on alterations in hippocampal PV-immunoreactivity (Table C in Supplementary Table S2), it was observed that both factors significantly (*p* < 0.01) affected PV expression in all investigated regions (and the total surface) of the hippocampus. However, the analysis revealed that the impact of the AASs protocols was greater (η^2^ values ranging from 0.464 for the CA1 to 0.858 for the total section) compared to the effect of prolonged exercise protocol (η^2^ values ranging from 0.103 for the CA1 to 0.665 for the total hippocampal sections).

### The Algorithm in the Relationships Between Hippocampal Sex Hormones Receptors and Behavioral Outcomes

**Figure 8:**
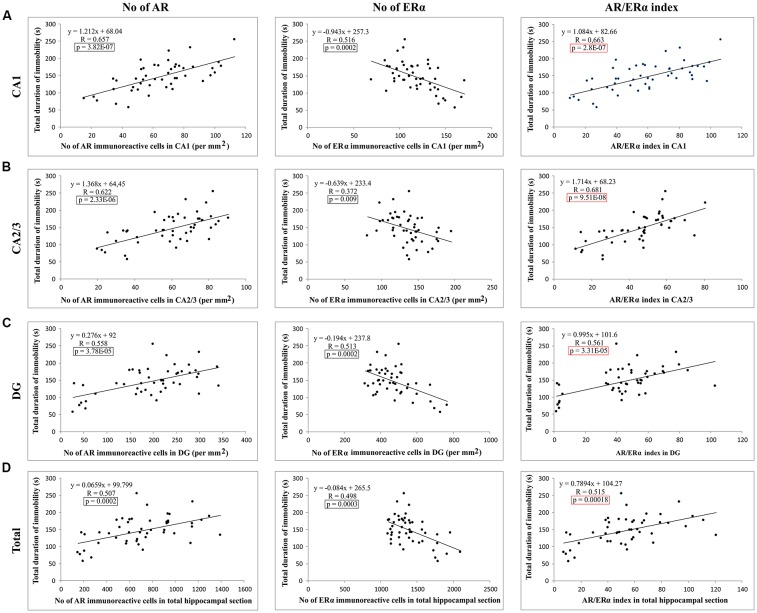
The relationship between the number of sex hormones receptors immunoreactive cells [ARs, estrogen receptors α – ERα and quotient of AR and ERα (AR/ERα index)] and the TDI in different regions of the hippocampus and total estimated hippocampal sections **(A)** CA1, **(B)** CA2/3, **(C)** DG, **(D)** total hippocampal section (*n* = 48). Simple regression analysis indicated that the number of AR and AR/ERα index strongly positively correlated with the TDI in all investigated regions and total estimated hippocampal sections. The number of ERα immunoreactive cells negatively, also significantly, correlated with TDI. The significant differences are framed (in black) and the highest significance is marked in red.

Evaluating the potential relationship between sex hormones serum levels and the results of behavioral testing (Table 1), a simple regression analysis confirmed no significant direct relationship between serum hormones levels and TDI. However, further analysis that estimated the interaction between behavioral manifestations and sex hormones receptors in the rat hippocampus revealed a significant correlation between AR and ERα expression, as well as the AR/ERα index, in the hippocampus and depressive-like behavior (Figure 8). The number of AR-immunoreactive cells in all regions strongly and positively correlated with increased depressive-like behavior (Figures 8A–C). This was also found to be the case for the total hippocampal sections (Figure 8D, *p* = 10^−7^–10^−4^, from the CA1 to total section). In contrast, the number of ERα immunoreactive cells in all hippocampal regions negatively correlated with the TDI (Figures 8A–D, *p* = 10^−4^–10^−3^). Finally, the simple regression analysis showed that the AR/ERα index had the strongest (positive) correlation with the same prodepressant marker obtained in the TST compared to the numbers of either AR or ERα (Figures 8A–D, *p* = 10^−8^–10^−4^).

**Table 1:** The relationship between sex hormones serum levels and the total duration of immobility (TDI; *n* = 48).

	Testosterone vs. TDI	DHT vs. TDI	Estradiol vs. TDI
Y	0.823x + 134.4	0.007x + 134.6	1.125x + 121.4
Pearson’s R	0.251	0.182	0.214
P	0.08	0.2	0.1

*Simple regression analysis indicated a weak correlation between testosterone, DHT, and estradiol levels with the TDI (Pearson’s r: 0.251, 0.182, 0.214, respectively)*.

**Figure 9:**
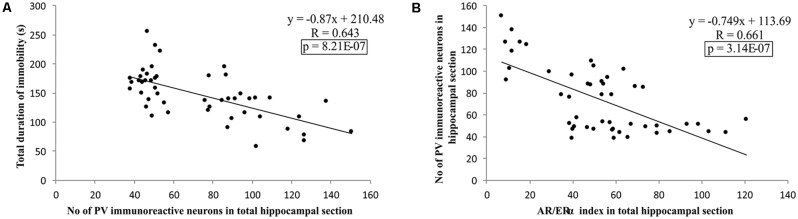
The relationship between the number of PV positive interneurons in total estimated hippocampal sections with the TDI **(A)**, and AR/ERα index **(B)** in total hippocampal sections (*n* = 48). Simple regression analysis indicated a strong negative correlation between the total number of PV interneurons with TDI and AR/ERα index with the number of PV positive interneurons.

Additionally, as shown in Figure 9, the results of this study confirmed that the number of PV immunoreactive neurons in the total hippocampal section strongly (negatively) correlated with the TDI, the principle prodepressant marker obtained from behavioral testing (*p* = 8.21*10^−7^). Finally, the simple regression analysis confirmed that the quantitative relationship between the hippocampal AR and ERα, expressed as the AR/ERα index, significantly and negatively correlated with the number of PV immunoreactive neurons in the total hippocampal section (*p* = 3.14*10^−7^).

## Discussion

The protocols applied in this study significantly altered depressive-like behaviors, as determined by the TST. Although there are reports that confirm the antidepressant effects of AASs in mice (Frye and Walf, 2009) and rats (Wainwright et al., 2016), it is hard to compare these with the results obtained in this study due to fact that the protocols performed in the aforementioned studies were different, using much lower doses (even in a single dose immediately before the testing) on the animals with previously altered mechanisms of endogenous production, by means of aging or gonadectomy (i.e., under circumstances that are not adequate to mimic AASs effects of prolonged treatment in young and healthy subjects in the human population). The depressive-like effect of chronic treatment with both AASs at the supraphysiological doses observed in this study are in accordance with previously published reports showing the depressive-like effect of ND and stanozolol in trials performed on rats that lasted for two (Rainer et al., 2014) and four (Matrisciano et al., 2010) weeks, while that effect was not observed in mice (Ambar and Chiavegatto, 2009). A recent study by Ludwig et al. (2019) also revealed the prodepressant effect of AAS (testosterone-propionate) at a supraphysiological dose, when the animals faced an aversive stimulus, such as the one performed in our behavioral testing. A possible explanation for the behavioral alteration induced by AASs can be found in a study that confirmed the AASs-induced decline in serotonin and dopamine in specific brain regions involved in the regulation of depressive levels (Krishnan and Nestler, 2008). Furthermore, it has been reported that AASs reduce hippocampal levels of neurotrophic growth factors (Tirassa et al., 1997), including BDNF (Krishnan and Nestler, 2008), with a consequent decline in neurogenesis (Tirassa et al., 1997) and hippocampal volume, a phenomenon that might be involved in the pathogenesis of depression (Krishnan and Nestler, 2008). The antidepressant effect of the prolonged (erobic) exercise protocol used in this study was obvious, compared to all sedentary groups (with or without AASs treatment). Similar results related to the antidepressant effect of chronic exercise were reported for mice (Duman et al., 2008) and rats (Greenwood et al., 2003), as well as in clinical trials (Fox, 1999). The proposed mechanisms for the antidepressant action of exercise are predominantly connected to increased BDNF content in the hippocampus (Bjørnebekk et al., 2005) that may account for enhanced neurogenesis and diminished clinical manifestations of depression (Leite et al., 2012), with a significant role of β-endorphins (Dinas et al., 2011). The exercise-induced increase in neurogenesis may also involve augmentation of endocannabinoids levels (De Moor et al., 2006), as well as alterations in hypothalamic-pituitary-adrenal axis with elevated ACTH release and lowered cortisol levels (Wittert et al., 1996).

As shown in Figure 3, all applied protocols significantly increased serum levels of T. The AASs-induced increase in T concentrations observed in this study is in line with the reported elevation of serum T following both acute (Minerly et al., 2010) and chronic (Takahashi et al., 2004; Zhang et al., 2016) administration of various AASs, which may be a consequence of up-regulated endogenous production in Leydig cells (Pomara et al., 2016). Interestingly, the exercise protocol, as well as chronic treatment with ND, did not significantly increase serum DHT levels, while TE administration in both sedentary and exercise groups resulted in elevated DHT levels in serum. Our results are in accordance with the previously reported insufficiency of ND to elevate DHT levels (Takahashi et al., 2004). Interestingly, Okamoto et al. (2012) also reported that mild exercise did not alter plasma DHT levels, but significantly increased DHT levels produced in the hippocampal neurons probably due to increased 5-α reductases mRNA expression in the hippocampus. Since we did not obtain data for endogenous hippocampal sex hormones production in this study, it seems that this unequal response to exercise in terms of DHT levels may be important for better understanding the role of local sex hormones actions in the hippocampus. Neither prolonged exercise nor chronic AASs administration, significantly affected serum E2 levels in this study. Previous studies offer different effects of various AASs on sex hormones levels. T application did not significantly affect serum E2 levels (similar to the results obtained in this study), but stanozolol administration decreased, while ND treatment increased serum E2 levels in rats (Lumia and McGinnis, 2010). This confirms that the response of sex hormones levels following AASs treatment strongly depends on the chemical structure of those compounds, as well as on the applied dose and treatment duration. However, the evaluation of the impact of AASs on sex hormones levels is even more complex, since both applied AAS are metabolized into active forms of sex hormones (Clark and Henderson, 2003). Both TE and ND can be transformed into DHT (by the action of 5α-reductase), and TE (like other testosterone esters) can also be aromatized into E2, while ND may be metabolized in the same manner, but only with 20% efficiency compared to TE (Ryan, 1959; Winters, 1990).

Unlike the PV (Figure 1), the immunoreactivity for both investigated sex hormones receptors was mostly found in pyramidal cells, with a very discrete presence in the molecular layer (Supplementary Figure S1). Both the prolonged exercise and chronic AASs protocols significantly affected the number of hippocampal AR immunoreactive neurons, but in the opposite directions. While the swimming training reduced AR-immunoreactivity in all investigated hippocampal regions, as well as in the total hippocampal section, ND and TE significantly increased the number of ARs. The potentiation of AR expression in the hippocampus induced by AASs in this study was obvious in both sedentary animals and those that exercised. The results obtained in this study are in line with the previously reported increase in the number of AR immunoreactive cells in the hippocampus of gonadectomized rats following intracerebroventricular injection of testosterone (Moghadami et al., 2016), as well as the intact rat hippocampus after treatment with AASs cocktail—testosterone cypionate, ND, and boldenone undecylenate (Menard and Harlan, 1993). However, the precise mechanism of AASs supplementation on hippocampal AR is still to be investigated. Meanwhile, it seems that the alterations in hippocampal BDNF content may significantly contribute to this process (Atwi et al., 2016). Based on the results of a study that analyzed the impact of physical activity on AR expression in the hippocampus following 2 weeks of mild erobic exercise, it is likely that the exercise-induced alterations in the number of hippocampal ARs may involve changes in AR mRNA levels with simultaneous alterations in neurogenesis (Okamoto et al., 2012). The alterations in neurogenesis (quantified in that study by means of proliferation, differentiation, and neuronal survival) might also explain the increased number of hippocampal ERα immunoreactive cells (Okamoto et al., 2012), such as observed in this study (Figure 5). The elevation in ERα-immunoreactivity was significant in the whole hippocampal sections, with the exception of the CA2/3 region. The observed increase in ERα immunoreactivity following exercise may be a consequence of altered hippocampal P450 aromatase mRNA expression, which augments locally synthesized E2 levels with consequent up-regulation of ERα in the hippocampus (Okamoto et al., 2012). On the other hand, the chronic administration of AASs did not significantly affect hippocampal ERα immunoreactivity.

Since it is well known that both hippocampal AR (Zuloaga et al., 2008) and ERα (Walf and Frye, 2006) may significantly affect mood, we concluded that, due to their usually opposing behavioral effects (Walf and Frye, 2006; Cunningham et al., 2012), defining and quantifying the AR/ERα index in the hippocampus seems to be meaningful in the context of behavioral alterations. Analyzing the impact of the performed protocols on the AR/ERα index, we noticed that exercise significantly lowered the index value in all investigated hippocampal regions, as well as in the total hippocampal section, while the supraphysiological doses of ND and TE administered for 6 weeks increased the AR/ERα index values (Figure 6). Interestingly, the AR/ERα index values correlated to TDI, and this correlation was even stronger than the correlations with the number of either hippocampal AR or ERα individually (although serum levels of sex hormones did not significantly correlate to TDI, Table 1) in all three hippocampal regions and the total section (Figure 8).

Similar to the results for the effects of a supraphysiological dose of ND obtained in our previous study (Selakovic et al., 2017), the chronic AASs treatment performed in this study also decreased the number of hippocampal PV immunoreactive interneurons, with the most prominent action in the DG for both ND and TE (Figure 7). Due to the fact that there are no published results considering the effects of AASs supplementation on PV immunoreactivity in the hippocampus of intact animals, we are only able to confirm that the results obtained in this study are connected to the previously reported decline in hippocampal plasticity following the application of AASs containing implants in gonadectomized male rats (Harley et al., 2000). The observed elevation in hippocampal PV immunoreactivity after completion of the extensive exercise protocol in this study is in accordance with the results previously obtained with the same exercise protocol (Selakovic et al., 2017), as well as with the results reported in trials performed on adult (Arida et al., 2004; Nguyen et al., 2013) and in the adolescent (Gomes da Silva et al., 2010) rats. The impact of exercise on the hippocampal formation of the GABAergic system has been attributed to specific cell proliferation and neurogenesis (Arida et al., 2004), although it still remains unclear whether it is *de novo* neurogenesis or if these processes occur in already existing cells (Eilam et al., 1999).

The results of our study showed a strong and negative correlation between the number of hippocampal PV-immunoreactive neurons and depressive-like behavior in the TST (Figure 9), confirming the essential role of the hippocampal GABAergic inhibitory system in the control of -like behavior/symptoms observed in preclinical (Shen et al., 2012) and clinical (Luscher et al., 2011) studies. However, even the stronger correlation between the AR/ERα index in the hippocampus and the number of hippocampal PV immunoreactive neurons (Figure 9) explicitly suggests the potential role for a quantitative relationship between the two sex hormones receptors (with the opposing behavioral actions) in the regulation of the depressive-like behavior *via* the hippocampal GABAergic system in rats. Therefore, it seems reasonable to consider this ratio as a potential new (and advanced) tool for the evaluation of behavioral alterations that involve changes in hippocampal sex hormones receptors, since it offers better insight than the analysis of each individual receptor alone.

## Data Availability

All datasets generated for this study are included in the manuscript and the supplementary files.

## Author Contributions

Tasks of individual authors: DS, JJ, DM and GR: conceptualization. DS, JJ, NJ, SM, VM, JK, NM and GR: data curation and investigation. DS, JJ, NJ, DM, SP and GR: formal analysis. DS and GR: funding acquisition and resources. DS, JJ, NJ, SM, VM, JK and GR: methodology. DS, JJ, NJ, SM, VM, JK, DM, SP and GR: writing.

## Conflict of Interest Statement

The authors declare that the research was conducted in the absence of any commercial or financial relationships that could be construed as a potential conflict of interest.

## Supplementary Material

FIGURE S1 The representative photomicrographs for the most prominent effects of exercise and AASs on evaluated hippocampal immunoreactivity.

TABLE S1 The raw data for the parameters obtained in this study.

TABLE S2 Two-way ANOVA of independent and joint effects of estimated variables for parameters obtained in this study.
