# Supplementary figures and images for: The Impact of Hippocampal Sex Hormones Receptors in Modulation of Depressive-Like Behavior Following Chronic Anabolic Androgenic Steroids and Exercise Protocols in Rats
Source: Front Behav Neurosci. 2019 Feb 7;13:19. doi: 10.3389/fnbeh.2019.00019 (PMC6374347; doi:10.3389/fnbeh.2019.00019)

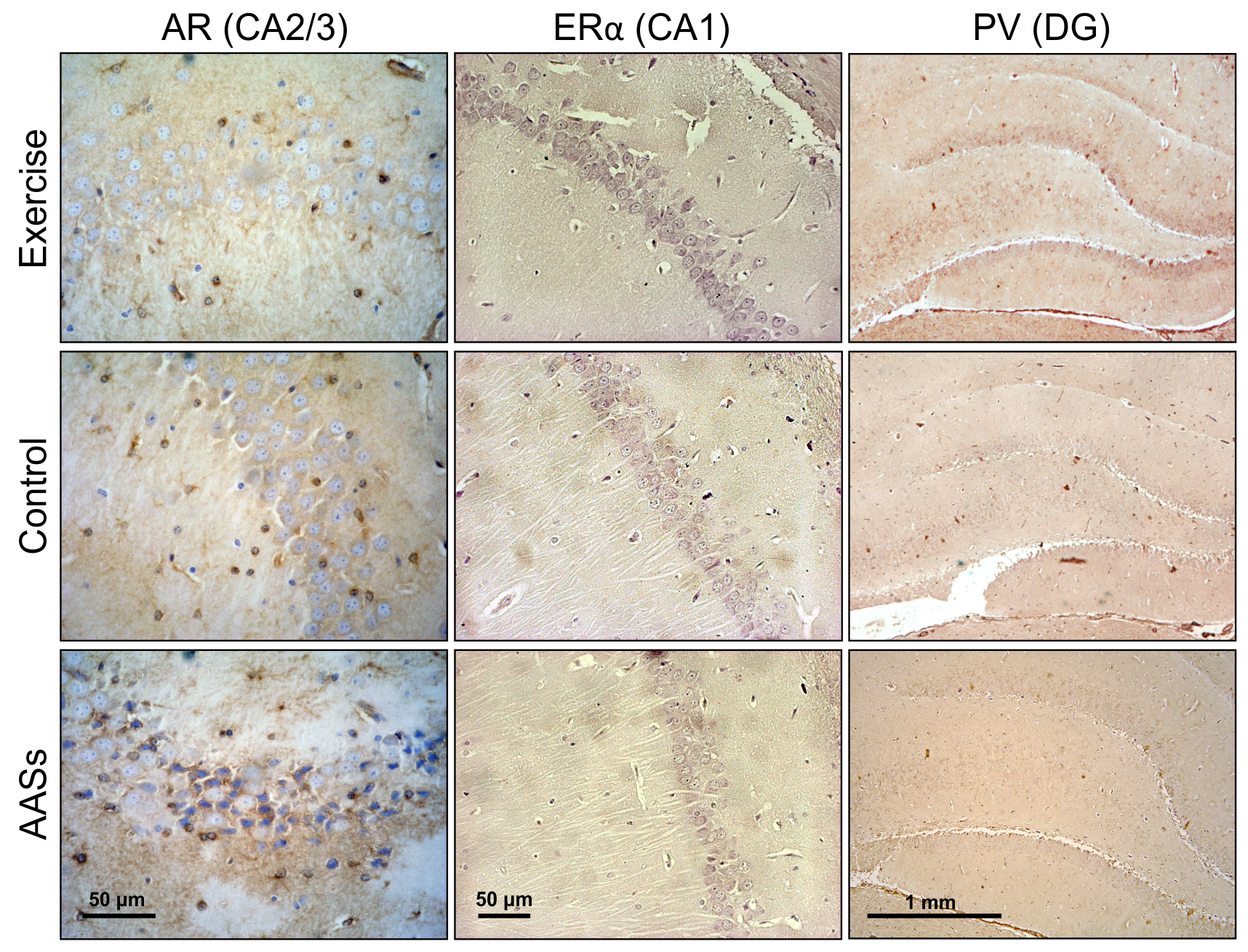

Supplement: FIGURE S1 — The representative photomicrographs for the most prominent effects of exercise and AASs on evaluated hippocampal immunoreactivity. [file Image_1.TIF]
